# Evaluation of a Patient CAM-with-Chemotherapy Educational Brochure

**DOI:** 10.1155/2015/408430

**Published:** 2015-02-23

**Authors:** Peter J. Smith, Alexandra M. Clavarino, Jeremy E. Long, Kathryn J. Steadman

**Affiliations:** ^1^School of Pharmacy, The University of Queensland, Brisbane, QLD 4072, Australia; ^2^Sunshine Coast Cancer Care Services, Nambour General Hospital, Nambour, QLD 4560, Australia

## Abstract

Biologically active CAM may detrimentally interfere with chemotherapy treatment, so cancer patients require targeted, evidence-based information on chemotherapy-CAM integration consequences. The object of this study was to investigate the potential for medical doctor recommendation and patient acceptance of a purpose-designed patient educational brochure on the safe use of CAM with chemotherapy. Cancer care doctors (*n* = 17) were provided a draft version of a patient educational brochure developed by the authors and completed a structured feedback form. Cancer patients receiving treatment (*n* = 12) were provided with the brochure and completed the local health service consumer testing feedback form. All 17 doctors perceived a need for the brochure and all would recommend the brochure to their patients. Approximately 59% of the doctors indicated they would recommend the brochure to all patients receiving chemotherapy and 41% preferred that only patients using CAM or who enquired about CAM be given the brochure. Cancer patients receiving chemotherapy reported that the brochure information answered their questions and was easy to understand. This evidence-based CAM-chemotherapy patient brochure may be a useful adjunct for use by cancer care health professionals to educate patients on the potential dangers of biologically active CAM use with chemotherapy and to provide patients with safe CAM alternatives.

## 1. Introduction

The majority of patients receiving chemotherapy will consider taking complementary and alternative medicine (CAM) [1] at some time during their treatment. CAM is defined as a broad and diverse group of treatments and products that do not tend to be widely used by conventional healthcare professions [2]. Biologically active CAM have the potential to interact with conventional medicines, including antineoplastic treatments [3] and herbal CAM interactions with chemotherapy have been estimated to be responsible for a substantial number of unexpected toxicities and possible undertreatment of some cancers [4]. Studies on patients receiving chemotherapy have concluded that one-quarter of patients taking biologically active herbal and/or vitamin supplemental CAM are at risk of a clinically relevant interaction [5, 6].

Mind-body CAM has shown efficacy in supportive care for patients receiving chemotherapy and when applied by appropriately trained therapists, using reasonable patient specific precautions, it is safe to use at that time [7]. Mind-body therapy CAM is a safe option for cancer patients who wish to use CAM during chemotherapy.

Patients would like to receive specific information on which CAM is safe to use with chemotherapy before their treatment commences [6]. The purpose of this study was to evaluate the acceptance by doctors and consumers of an evidence-based brochure designed to provide answers to common questions chemotherapy patients have about CAM use with chemotherapy.

## 2. Methods

An educational brochure was developed by the authors (see Appendix in Supplementary Material available online at http://dx.doi.org/10.1155/2015/408430) with the aim of providing a tool for use by cancer care doctors and associated cancer health professionals to give evidence-based guidance to patients on CAM use with chemotherapy. Eighteen doctors at the Sunshine Coast Cancer Care Service (Nambour Hospital, Queensland, Australia) were asked to provide feedback concerning the draft patient brochure through completing a structured feedback form. One doctor did not return the feedback form. Six oncologists, 2 oncology training registrars, 5 haematologists, 2 haematology training registrars, and 2 general rotational hospital registrars gave written feedback. Following doctor feedback, the draft brochure was submitted to the local health service publishing unit for publication. The relevance, clarity, presentation, readability, and acceptance of the brochure were tested through distribution to 12 chemotherapy patients (Table 1) who each completed the Sunshine Coast Hospital and Health Service consumer testing feedback form.

## 3. Results

### 3.1. Doctor Feedback

All 17 doctors thought there was a need for the brochure and responded that they would recommend the brochure to their patients (Table 2). Approximately 59% of doctors said they would recommend the brochure to all patients receiving chemotherapy, but 41% preferred that only patients using CAM or asking about CAM be given the brochure. Two doctors preferred to personally give the educational CAM brochure to the patient, 7 at nurse education clinic, 3 by the doctor or at nurse clinic, and 5 by doctor, nurse clinic, or other cancer care health professional. No doctors sought to limit the brochure to early stage/curative intent patients.

Feedback received from doctors on the brochure wording and/or phrasing, when thought to be appropriate by the authors, was incorporated into the brochure. A haematologist indicated that it* “May be worth noting that ‘invasive' mind-body techniques (eg acupuncture) could be an issue in [a] setting of low platelets, and so forth”* but did note the limitation for such details within the brochure's scope and intended broad message. Another clinician was concerned:* “some fish oils have antiplatelet effect and could be an issue with haematology patients with low platelets.”* A fish oil caution was not included in the brochure because at risk patients are closely monitored, the antiplatelet effect is not great (10 g of fish oil per day has less effect on platelet function than 100 mg of aspirin given intravenously [8]), and dietary fish oil may be consumed in amounts similar to standard supplements. One doctor sought the inclusion of B group vitamins and vitamin E for the treatment of peripheral neuropathy; however, a 2008 Cochrane review concluded insufficient evidence for the use of vitamin B [9], and vitamin E may have chemotherapy interaction complications [10]. Another prescriber's suggestion of adding grapefruit prohibition was not considered within the brochure brief.

Doctors were generally positive when optional extra feedback was given:

*I think it is just right- short and balanced*

*Overall, great idea and good brochure*

*Good amount of writing. Messages are clear*

*Well put together brochure. Definitely useful*

*Excellent idea. I liked it. So many patients ask questions which this brochure is going to answer*

*Definitely a topic poorly understood by many patients and doctors/health care professionals. Patients often ask about the benefits/risks associated with CAMs during chemo. This brochure will help*

One doctor criticized the brochure for being too academic:* “Needs editing for English-it's written in academic English, needs to be properly edited for patient reading”*. However, the brochure was considered to be appropriate by the authors and so few changes were made; the brochure was instead submitted for publication and subsequent consumer testing to determine if it was understandable.

### 3.2. Patient Feedback

Patients were generally enthusiastic about the brochure and all 12 found the information clear and easy to understand. All patients thought that the information was relevant to them and that the brochure contained enough information to answer their CAM questions (Table 3).

*It's a great little book- a good size that you would read because it is not over- involved. It's matter-of-fact and gives you what you need to know in an easily understandable read. I think I would've found this booklet very beneficial when I started my regime*

*To all concerned with the research of the publication of this information- well done*

*I found it a great and informative book: clear and to the point, thank you*

Additional patient feedback indicated that the main messages in the brochure, that is biologically active CAM may interact with chemotherapy and should be declared to conventional health professionals, were understood:

*I would certainly ask more questions of the nursing staff/GP/specialist*

*Just check with your specialist first to make sure it's not going to have adverse reaction with the chemo*

* Talk to your doctor, ask questions about CAM, tell your doctor if you are taking any CAM*

*I was unaware that using “natural” remedies antioxidant, herbs could be counter productive*

The brochure was also effective in making patients aware that CAM therapies are effective and safe to use for support during chemotherapy treatment:

*Complementary therapies can work*

*Good to know about the yoga and other things which won't affect the chemo but could be substantially helpful with your wellbeing*

*Would certainly look into more of the massage, reflexology, and so forth, also the meditation and yoga*

## 4. Discussion

The evidence-based educational brochure was designed to be brief and as neutral as possible, neither supportive of, or against CAM use. The authors deemed this necessary to not alienate potential CAM users who may be receiving alternative CAM advice [7]. Chemotherapy patients are likely to be contending with CAM advice from a variety of sources including family, friends, practitioners, and even casual acquaintances, who may be particularly insistent and persuasive when they regard themselves as having CAM expertise [1, 6, 11, 12]. Conventional health practitioners need to provide evidence-based CAM information to chemotherapy patients to counter CAM misconceptions patients may have and to guide patients away from taking CAM which may be potentially detrimental to their treatment.

All cancer care doctors indicated they would use the brochure for their patients. A proportion of doctors wanted only patients asking about or using CAM to receive the brochure. These doctors were concerned that giving CAM information to patients may be misconstrued as being promotional of CAM use with chemotherapy and one patient did report she would look into more mind-body CAM and supplements that may help if she were able to take them, as a result of reading the brochure. If only patients who ask about CAM were offered the brochure, patients in need of CAM guidance with chemotherapy may be missed; cancer patients do not necessarily volunteer their CAM consumption unless asked [3], prefer their health care providers to initiate discussions regarding CAM use [13], want safety information regarding CAM with chemotherapy before they start treatment [6], and make CAM decisions at the same time as standard medical decisions [11].

Though there are existing general information resources on CAM use, chemotherapy patients want to receive specific evidence-based information on CAM use at the time of receiving chemotherapy [6]. This small population study demonstrated that a brochure showed promise in providing cancer patients' educational requirements on the safe use of CAM with chemotherapy and may be a useful tool for use by cancer care health professionals to educate patients on potential dangers of biologically active CAM use with chemotherapy and to provide patients with safe CAM alternatives if required.

## 5. Conclusion

The evidence-based CAM-chemotherapy patient brochure developed herein may be a useful adjunct for cancer care health professionals to educate patients on the potential dangers of biologically active CAM use with chemotherapy and to provide patients with safe CAM alternatives.

## Supplementary Material

This is an educational brochure developed by the authors, designed for use by cancer care doctors and associated cancer care health professionals, to give evidence-based guidance to patients on complementary and alternative medicine (CAM) use with chemotherapy.

## Figures and Tables

**Table 1:** Demographic characteristics of the chemotherapy patients surveyed (*n* = 12).

Characteristics	*n*
Age, years	
31–40	1
41–50	4
51–60	2
61–70	5
Sex	
Female	7
Male	5
Highest level of education	
Left school before year 10	1
Secondary (year 10)	4
Secondary (year 12)	3
Tertiary	4

**Table 2:** Doctor feedback with breakdown in terms of medical specialty.

	Oncologist *n* = 6		Haematologist *n* = 5		Oncology registrar *n* = 2		Haematology registrar *n* = 2		Rotational registrar *n* = 2		Total *n* = 17	
	Yes	No	Yes	No	Yes	No	Yes	No	Yes	No	Yes	No
Do you think there is a need for this brochure?	6	—	5	—	2	—	2	—	2	—	17	—
Would you recommend this brochure to your patients?	6	—	5	—	2	—	2	—	2	—	17	—

Which patients receiving chemotherapy would you recommend this brochure to? (more than one answer if required)												
All patients	4		3		1		2		1		10	
Patients/carers who ask about CAM or using CAM	3		2		1		0		1		7	
Only early stage/curative intent patients	—		—		—		—		—		—	
Other (specify)	—		—		—		—		—		—	

Who should give this brochure to the patient? (more than one answer if required)												
Treating consultant/registrar	4		2		2		0		1		9	
Nurse clinic	4		5		2		2		2		14	
Other cancer care professionals (specify)	1 (pharmacist)		1 (any)		1 (pharmacist)		0		1 (any)		4	
General practitioner	0		0		0		0		1		1	
Other (specify)	1 (any health practitioner that finds out patient using CAM)		0		0		1 (with other handouts in patient pack)		0		2	

**Table 3:** Patient feedback, with breakdown in terms of gender and patient malignancy (Haem = haematology; Oncol = oncology) and numbers indicating those who answered yes (Y), no (N), or did not answer the question (na).

	Total *n* = 12		Female *n* = 7		Male *n* = 5		Haem *n* = 4		Oncol *n* = 8	
	Y	N/na	Y	N/na	Y	N/na	Y	N/na	Y	N/na
Did you find the information clear, easy to read and understand?	12	0/0	7	0/0	5	0	4	0/0	8	0/0
Was there enough information to answer your questions?	12	0/0	7	0/0	5	0	4	0/0	8	0/0
Did the information explain medical terms well?	12	0/0	7	0/0	5	0	4	0/0	8	0/0
Do you believe this information is relevant to you?	12	0/0	7	0/0	5	0	4	0/0	8	0/0
Is the content culturally sensitive and appropriate?	10	0/2	5	0/2	5	0	3	0/1	7	0/1
Is the design appropriate and appealing?	12	0/0	7	0/0	5	0	4	0/0	8	0/0
Is the design clear, uncluttered, and easy to follow?	12	0/0	7	0/0	5	0	4	0/0	8	0/0
Is the print large and clear enough?	12	0/0	7	0/0	5	0	4	0/0	8	0/0
Did the photos and illustrations help you to understand the information?	11	0/0	6	1/0	5	0	3	1/0	8	0/0
