# Supplementary material for: Evaluation of a Patient CAM-with-Chemotherapy Educational Brochure
Source: Evid Based Complement Alternat Med. 2015 Feb 23;2015:408430. doi: 10.1155/2015/408430 (PMC4352944; doi:10.1155/2015/408430)
Supplement: Supplementary file 1 — This is an educational brochure developed by the authors, designed for use by cancer care doctors and associated cancer care health professionals, to give evidence-based guidance to patients on complementary and alternative medicine (CAM) use with chemotherapy. [file 408430.f1.pdf]

What complementary and alternative medicine (CAM) can I safely use with chemotherapy?

Evidence-based information for patients

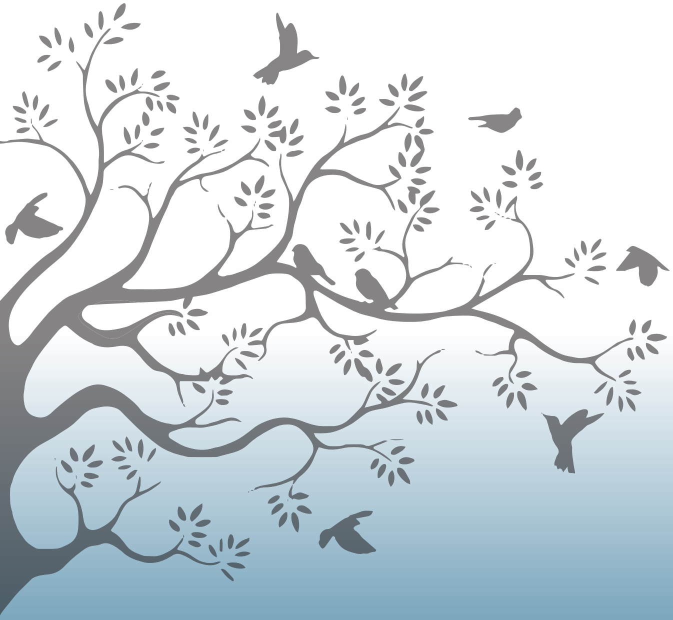

Version 1.0.042014

Peter J Smith,[1,2] Alexandra Clavarino,[1] Jeremy Long[2] and Kathryn J Steadman[1]
[1]University of Queensland, Brisbane, Australia
[2]Sunshine Coast Cancer Care Services, Nambour, Australia

Introduction

This booklet is designed to advise you on what we currently know about complementary and alternative medicine (CAM) use at the time of receiving chemotherapy. It does not cover CAM use at other times, for example, when you have completed conventional treatment.
It is not a criticism of CAM use, just a summary of current evidence-based studies on CAM effectiveness and safety at this time.

What is CAM?

Complementary and alternative medicine (CAM) are products and treatments which are not widely used by conventional healthcare professionals.[1] CAM that is absorbed into the bloodstream, such as herbs and vitamin supplements, may interfere with chemotherapy effectiveness.[2,3]

Mind-body CAM

Mind-body CAM such as massage, meditation, music, imagery, hypnosis, self-expression, mild exercise and acupuncture have established evidence and are able to be used safely with standard treatment.
Mind-body therapies should be your first consideration when looking for safe, complementary cancer care. (Table 1)

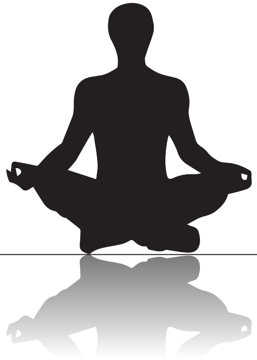

This booklet will provide you with evidence-based information on which CAM we know is safe for you to use, if you wish, during treatment with chemotherapy.

Herbs and vitamin supplements are absorbed into the bloodstream and may interfere with chemotherapy effectiveness.

Mind-body therapies are safe and effective complementary treatments you can use while receiving chemotherapy.

Table 1.

Safe, evidence-based mind-body CAM that may help during chemotherapy treatment[4]

* Acupuncture, hypnosis and massage should be administered by specially trained practitioners

Mind-body therapy CAM	Supportive care use
Acupuncture*	• decreases acute vomiting caused by chemotherapy
Hypnosis*	• decreases nausea and vomiting caused by chemotherapy
Imagery and relaxation (body relaxation combined with visualisation e.g. imagining warm sun melting away cancer cells)	• boosts immune functioning during treatment
Massage*	• decreases nausea and vomiting caused by chemotherapy<br>• reflexology (foot massage) decreases anxiety during treatment
Meditation (combines relaxation with self-awareness, which brings the person into the moment, free from anxiety concerning the past or future)	• alters immune patterns by decreasing stress<br>• decreases anxiety and depression
Music	• reduces anxiety
Self-expression (includes written or verbal expression, artwork, humour and movement)	• decreases dark feelings and improves outlook
Mild exercise (includes yoga)	• reduces fatigue and promotes wellbeing

Other mind-body CAM that is safe to use during chemotherapy treatment: Aromatherapy (inhaled), Bowen therapy, Qi gong, Reiki, Tai Chi, Therapeutic touch

Is there evidence to use CAM?

To treat cancer

There is not yet enough conclusive evidence to recommend to you any CAM to treat cancer. Delaying conventional treatment to use CAM alone to try and treat cancer may potentially sacrifice years of your life, particularly if you are being treated with curative intent.[5]

For chemotherapy support

Apart from mind-body therapies (Table 1) and ginger for the treatment of nausea, it is very difficult to find consistent, high quality studies which show beyond doubt that a CAM is effective, is safe, and is able to be recommended to you when you are receiving chemotherapy. Probiotics have shown some benefit to lessen diarrhoea, which can be a side effect of particular chemotherapy and fish oil has helped some lung cancer patients resist weight loss (Table 2).

Table 2.

Oral CAM that may help during chemotherapy treatment[4]

Oral CAM	Supportive care use
Ginger	• reduces nausea caused by chemotherapy
Probiotics/ Yoghurt	• decreased diarrhoea caused by chemotherapy<br>**Caution**<br>Probiotics are normally well tolerated; however, there have been rare cases of very ill patients receiving chemotherapy developing serious probiotic-caused infections
Fish oil supplements	• fish oil taken daily may provide benefit to patients with lung cancer through maintenance of weight during chemotherapy administration

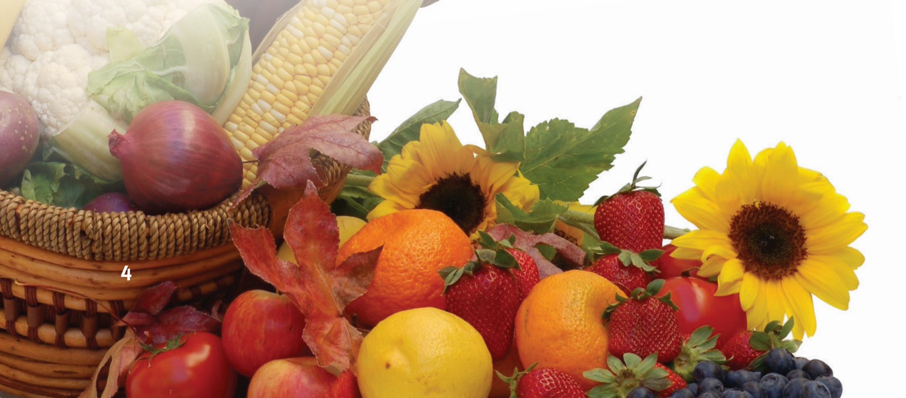

Already using CAM to treat something else?

You may already be taking herbal medicines or vitamin supplements regularly to treat existing conditions, or occasionally for example to treat cold symptoms. It is important that you declare this CAM use, as there may be a chance of interaction depending on the chemotherapy you are receiving.[6]

How may CAM interfere with chemotherapy?

Practitioners carefully calculate chemotherapy doses individually for you, as accuracy is crucial in ensuring that you get the maximum anticancer action with the least side effects possible. CAM that are absorbed into the bloodstream, such as herbal medicines, may potentially interfere with your chemotherapy in a way that lessens the anticancer effect or gives you more side effects by making the chemotherapy more toxic to you.[2,3] (Table 3)

Potential interactions with CAM may make chemotherapy more toxic to you, or may lessen the anticancer effect of the chemotherapy.

Antioxidants: can I take them?

The use of supplemental antioxidants by cancer patients receiving chemotherapy remains controversial. Beneficial effects of taking antioxidant supplements to reduce side effects may be at the cost of lessening the chemotherapy anticancer effect, depending on which chemotherapy you are receiving[7] (Table 3). Until further definitive research is done, caution is advised when taking antioxidant supplements at the time of receiving chemotherapy.[8]

Beneficial effects of taking antioxidants to reduce side effects may be at the cost of lessening the chemotherapy anticancer effect

Fortunately, eating antioxidant-containing foods in your normal diet is safe with chemotherapy.[7]

Antioxidant-containing foods in your normal diet do not achieve high enough levels to interact with chemotherapy.

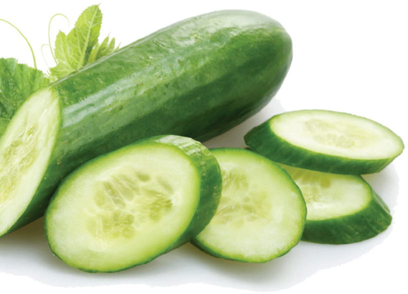

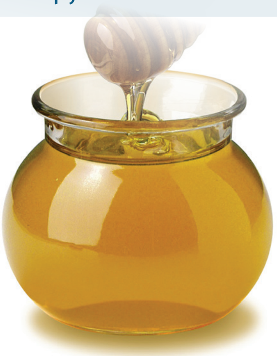

Table 3.

CAM and potential interactions with chemotherapy[2,3,7,9,10]

CAM	Interaction with chemotherapy	Explanation
Antioxidant supplements (Vitamins A, C & E, CoQ10 and others)	Interaction likely with particular chemotherapy<br><br>Diminishes radiotherapy effectiveness	Concern that antioxidant supplements may protect cancer cells as well as normal cells
Herbs, Chinese herbs, Medical herbal teas	It has been estimated that herbal interactions are responsible for a substantial number of unexpected toxicities of chemotherapy and possible under treatment seen in cancer patients<br><br>Chinese herbal mixtures often contain many different plant extracts which are highly biologically-active, increasing chance of interaction	There is indirect evidence for both positive and negative effects of herbal use with chemotherapy; however, currently, there is not enough information available about herb-chemotherapy interactions to make definite recommendations
Medicinal mushrooms (*Coriolus versicolor* "PSK", *Ganoderma lucidum* "Reishi")	Interaction unlikely with most chemotherapy	No reported negative interactions
Trace mineral supplements	Interaction unlikely with very small doses of minerals	No information or valid explanations available for potential negative interactions
Homeopathy (also Bach flower remedies, Australian bush flower essences, Schuessler's tissue salts)	Interaction unlikely	Homeopathic products are so diluted that there is little chance of interaction

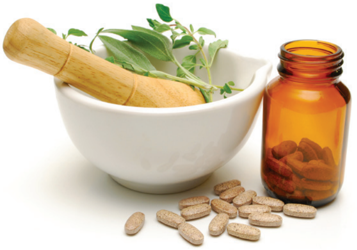

I still want to take CAM

If you decide to take CAM at the time you are receiving chemotherapy, for example if you have an advanced cancer and are willing to accept potential interaction risk, it is still very important that you tell your cancer specialist or pharmacist for documentation, to enable the best care to be given to you.

Require more information?

National Cancer Institute
Complementary and alternative medicine in cancer treatment:
http://www.cancer.gov/cancertopics/pdq/cam/cam-cancer-treatment/patient

Thinking about CAM: A guide for people with cancer
http://www.cancer.gov/cancertopics/cam/thinking-about-CAM

Memorial Sloan-Kettering Cancer Center
Complementary therapies to ease the way during cancer treatment and recovery
http://www.mskcc.org/cancer-care/patient-education/resources/complementary-therapies-ease-way-during-treatment-and-recovery

Cancer Council Australia
Complementary and alternative therapies:
http://www.cancer.org.au/about-cancer/treatment/complementary-therapies-and-cancer.html

References

1. Zollman C, Vickers A. ABC of complementary medicine. Complementary medicine and the patient. *BMJ* Dec 4 1999;319(7223):1486-1489.
2. Tascilar M, de Jong FA, Verweij J, Mathijssen RH. Complementary and alternative medicine during cancer treatment: beyond innocence. *Oncologist.* Jul-Aug 2006;11(7):732-741.
3. Meijerman I, Beijnen JH, Schellens JH. Herb-drug interactions in oncology: focus on mechanisms of induction. *Oncologist.* Jul-Aug 2006;11(7):742-752.
4. Smith PJ, Clavarino A, Long J, Steadman KJ. Why do some cancer patients receiving chemotherapy choose to take complementary and alternative medicines and what are the risks? *Asia Pac J Clin Oncol.* Mar 2014;10:1-10
5. Han E, Johnson N, DelaMelena T, Glissmeyer M, Steinbock K. Alternative therapy used as primary treatment for breast cancer negatively impacts outcomes. *Ann Surg Oncol.* Apr 2011;18(4):912-916.
6. Werneke U, Earl J, Seydel C, Horn O, Crichton P, Fannon D. Potential health risks of complementary alternative medicines in cancer patients. *Br J Cancer.* Jan 26 2004;90(2):408-413.
7. Ladas E, Kelly KM. The antioxidant debate. *Explore (NY).* Mar-Apr 2010;6(2):75-85.
8. Greenlee H, Hershman DL, Jacobson JS. Use of antioxidant supplements during breast cancer treatment: a comprehensive review. *Breast Cancer Res Treat.* Jun 2009;115(3):437-452.
9. Zeller T, Muenstedt K, Stoll C, et al. Potential interactions of complementary and alternative medicine with cancer therapy in outpatients with gynecological cancer in a comprehensive cancer center. *J. Cancer Res. Clin. Oncol.* Mar 2013;139(3):357-365.
10. Cheng CW, Fan W, Ko SG, Song L, Bian ZX. Evidence-based management of herb-drug interaction in cancer chemotherapy. *Explore (NY).* Sep-Oct 2010;6(5):324-329.

cc BY

© State of Queensland
(Department of Health) 2013
http://creativecommons.org/
licences/by/3.0/au/deed.en

Standard 1: Governance for Safety and Quality in Health Service Organisations
Involves setting direction, making policy and strategy decisions, overseeing and monitoring organisational performance and ensuring overall accountability for a service.

Standard 2: Partnering with Consumers
Consumers and/or carers provided feedback on this patient information.

Standard 4: Medication Safety
Reduction in the occurrence of medication incidents, and improvement to the safety and quality of medicine use.

Standard 11: Service Delivery
Patients and the community have access to safe, high quality healthcare services that are appropriate, effective and meet their needs.

Standard 12: Service Delivery
To ensure high quality care is delivered to consumers/patients through the care continuum.

Great state. Great opportunity.

Queensland Government
